# Comparative assessment of UV-C radiation and non-thermal plasma for inactivation of foodborne fungal spores suspension *in vitro*

**DOI:** 10.1039/d4ra01689k

**Published:** 2024-05-23

**Authors:** Markéta Kulišová, Michaela Rabochová, Jan Lorinčík, Olga Maťátková, Tomáš Brányik, Jan Hrudka, Vladimír Scholtz, Irena Jarošová Kolouchová

**Affiliations:** a University of Chemistry and Technology, Prague, Department of Biotechnology Technická 5, 166 28, Praha 6 Prague Czech Republic marketa.kulisova@vscht.cz; b Research Centre Rez, Department of Material Analysis Hlavní 130, 250 68, Husinec-Řež Czech Republic; c Czech Technical University in Prague, Faculty of Biomedical Engineering nám. Sítná 3105 272 01 Kladno Czech Republic; d Research Institute of Brewing and Malting Lípová 15 120 44 Prague Czech Republic; e University of Chemistry and Technology, Prague, Department of Physics and Measurements Technická 5, 166 28, Praha 6 Prague Czech Republic

## Abstract

Fungal contamination poses a persistent challenge to industries, particularly in food, healthcare, and clinical sectors, due to the remarkable resilience of fungi in withstanding conventional control methods. In this context, our research delves into the comparative efficacy of UV radiation and non-thermal plasma (NTP) on key foodborne fungal contaminants – *Alternaria alternata*, *Aspergillus niger*, *Fusarium culmorum*, and *Fusarium graminearum*. The study examined the impact of varying doses of UV radiation on the asexual spores of all mentioned fungal strains. Simultaneously, the study compared the effects of UV radiation and NTP on the metabolic activity of cells after spore germination and their subsequent germination ability. The results revealed that UV-C radiation (254 nm) did not significantly suppress the metabolic activity of cells after spore germination. In contrast, NTP exhibited almost 100% effectiveness on both selected spores and their subsequent germination, except for *A. niger*. In the case of *A. niger*, the effectiveness of UV-C and NTP was nearly comparable, showing only a 35% decrease in metabolic activity after 48 hours of germination, while the other strains (*A. alternata*, *F. culmorum*, *F. graminearum*) exhibited a reduction of more than 95%. SEM images illustrate the morphological changes in structure of all tested spores after both treatments. This study addresses a crucial gap in existing literature, offering insights into the adaptation possibilities of treated cells and emphasizing the importance of considering exposure duration and nutrient conditions (introduction of fresh medium). The results highlighted the promising antimicrobial potential of NTP, especially for filamentous fungi, paving the way for enhanced sanitation processes with diverse applications.

## Abstract 2

The comparative efficacy of UV radiation and non-thermal plasma (NTP) on key foodborn fungal contaminants was studied. UV-C radiation (254 nm) did not significantly suppress the metabolic activity of cells are spore germination whereas NTP exhibited almost 100% effectiveness.

## Graphical abstract

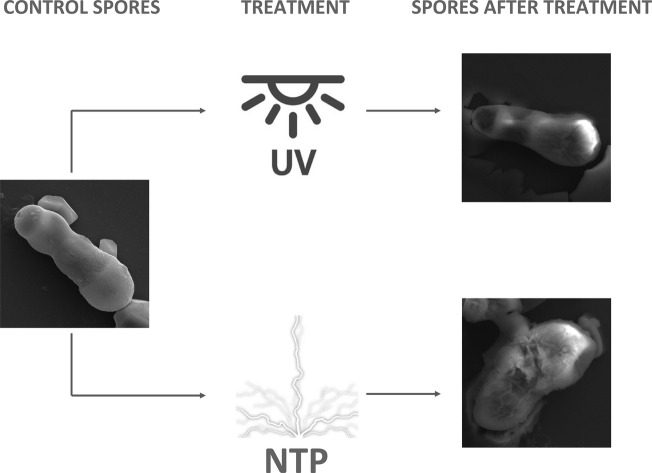

## Introduction

Fungi exhibit remarkable resilience as spoilage microorganisms, successfully circumventing the control methods employed by the food industry. Various types of fungal spores (asexual, sexual) are swiftly disseminated through water and air, exhibiting the ability to endure harsh environmental conditions and consistently multiply. Certain fungal species can endure even the most extreme physiochemical conditions and the rigorous thermal treatments employed in commercial food manufacturing. Yeasts and filamentous fungi excel in spreading and causing cross-contamination within food processing facilities. Thanks to their diverse structural characteristics and survival mechanisms, fungal species are exceptionally well-suited to survive in specific ecological niches, enabling them to contaminate and spoil food products (rice, maize, wheat, oilseed crops, fruits and fruit juices *etc.*).^1–3^ Fungal spores and biofilms, which exhibit enhanced mechanical and antimicrobial resistance, have become prominent areas of extensive research exploration.^4^

Common foodborne pathogenic fungi that are difficult to eliminate include the genera *Aspergillus*, *Alternaria* and *Fusarium*. The resistance of these genera increases the ability to produce toxins in secondary metabolism, especially the genera *Aspergillus* and *Fusarium*. In addition, spores of the genera *Aspergillus* and *Alternaria* contain pigments that also increase their resistance to elimination agents.^5,6^

Dry sanitation techniques^7^ or no-touch methods, including hot air, UV-C light, pulsed light, gaseous ozone (O_3_), and NTP, have the potential to enhance the effectiveness of traditional methods (chlorine-based solutions), leading to more efficient sanitization processes.^5,8,9^ Additionally, these approaches are environmentally friendlier than conventional wet methods, as they do not generate toxic waste such as acids and hydroxides usually employed in CIP processes, thus mitigating their environmental footprint.^7,10,11^ Furthermore, employing a combination of sanitation methods with distinct mechanisms of action presents a viable strategy to curtail the development of various types of microbial resistance against sanitation efforts and broaden the spectrum of antimicrobial effectiveness.^12^ Lately, there has been significant attention focused on various light-based approaches, which have shown their effectiveness in killing microbes, irrespective of their drug resistance, and are regarded as some of the most promising antimicrobial methods. These light-based therapies include treatments that have exhibited strong bactericidal activity in numerous laboratories and real-world studies: antimicrobial blue light^13^ (aBL; 400–480 nm), antimicrobial photodynamic inactivation^14^ (aPDI; 415 nm), pulsed light^15^ (PL; 500–1200 nm), and ultraviolet (UV) light (*e.g.* vacuum-UV^16^ (100–200 nm), Far-UV^17^ (200–230 nm), UV-C (200–280 nm), UV-B (280–320 nm), UV-A (320–400 nm)).^18,19^ As the efficacy of light is strongly dependent on the wavelength, light-based treatment can be targeted at various use cases over alternatives such as NTP, chlorine preparations, and thermal methods. UV radiation demonstrates significant effectiveness against bacterial and yeast spores or biofilms, as highlighted in multiple studies.^20,21^ In laboratory settings, it is common to expose surfaces to UV radiation.^22^ The accepted wavelength for use in the food industry is 254 nm.^23^ Irradiation times, however, vary widely for different microorganisms.^6–8^ Fungi exhibit a distinctive ability to not only survive but also thrive on surfaces with extremely low water activity (*a*_w_ = 0.6). Microorganisms capable of thriving on dry surfaces have developed an increased tolerance to various stressors, including UV radiation.^7^ These light-based treatments are considered low-risk in terms of the development of microbial tolerance or resistance due to their ability to target multiple cell structures, including cell envelopes, proteins, lipids, and genetic material, leading to deleterious effects.^24^

UV radiation is effective on various species, but the amount needed to be lethal varies significantly among different taxonomic groups. This variation is influenced by the specific characteristics of the microorganisms and various physical and chemical factors. UV radiation can also lead to mutagenic effects, particularly in lower, non-lethal doses. The destruction of microorganisms depends on the precise dosage of UV radiation applied.^25,26^ To provide perspective, achieving a survival rate of less than one percent requires specific UV radiation doses: 65 J m^−2^ for microorganisms of the genus *Shigella*, 129 J m^−2^ for *E. coli*, 129 J m^−2^ for the genus *Staphylococcus*, 2064 J m^−2^ for the genus *Fusarium*, and 4128 J m^−2^ for the genus *Aspergillus*.^27^ Microscopic filamentous fungi show higher resistance to UV radiation compared to bacteria and yeast. In the study by Tóthová *et al.*, species of the genus *Aspergillus* were found to be resistant to all tested doses of UV radiation. Their growth was ripened and even stimulated in some cases after exposure to UV radiation, changes in pigmentation were observed. When monitoring the ability of spore germination and growth after UV irradiation, the most resistant species were *Aspergillus nidulans*, *Aspergillus niger* and *Fusarium moniliforme*, which were able to grow even after 360 minutes of UV exposure (UV dose = 2548 J m^−2^).^26^ Cortesão *et al.* tested the effect of X-ray radiation, cosmic radiation and UV-C radiation on *Aspergillus niger* spore suspensions. In general, *Aspergillus niger* spores exhibited great resistance to elevated levels of X-ray radiation (LD_90_ = 360 Gy) and cosmic radiation (LD_90_ = 500 Gy for helium ions and 100 Gy for iron ions). Particularly noteworthy is their remarkable resilience to UV-C radiation, with an LD_90_ value of 1038 J m^−2^, a significantly higher tolerance compared to other radiation-resistant microorganisms such as *Deinococcus radiodurans*.^28^ In a study by Duque-Sarango *et al.*, the impact of UV radiation on *A. niger* and *Penicillium* spores in water was investigated. The effective inactivation dose immediately after UV treatment application was found to be 220.1 J m^−2^ for *A. niger* and 123.8 J m^−2^ for *Penicillium* sp. However, after 24 hours of photoreactivation, the required UV radiation dose for *Penicillium* spores inactivation increased by 53.8%.^25^ When investigating the impact of UV radiation on *Penicillium commune* and *Chaetomium globosum* present on historical tissue paper, the minimum dose of UV irradiation required to achieve fungicidal efficacy against all fungal contamination was estimated to be 118 J cm^−2^.^29^

To achieve fungal spore inactivation, UV radiation doses typically range from 500 to 36 000 J m^−2^ when sterilizing flow boxes. When sterilizing surfaces during operations, the dose typically falls within the range of 3000 to 20 000 J m^−2^.^27^ Despite the high tolerance of microscopic filamentous fungi to UV radiation, this method is still used as one of the most common sterilization methods in the food industry, pharmacy, *etc.* Thus the use of NTP could be a good alternative for the elimination of these microorganisms, since it is highly effective on bacterial and yeast cultures. NTP refers to a state of partially ionized gas characterized by the predominant storage of energy in free electrons, while maintaining a low overall temperature. Although there are fewer studies on the effect of NTP on microscopic fungi, NTP looks promising against microscopic fungi as well.^30–32^ The activity of NTP results in the generation of reactive oxygen and nitrogen species, causing damage to cell membrane integrity, disruption of energy metabolism, impairment of DNA and adverse effects on ion homeostasis.^33^

Hoppanová *et al.* provided a comprehensive overview of preliminary observations regarding the impact of NTP on fungi. In most experiments, growth reduction occurred after a specific post-harvest period, rather than complete microbial inactivation. NTP treatment durations varied from 5 minutes to 40 minutes, yet even at the longest duration, complete inhibition of fungal growth was not observed.^34^ Other publications have addressed the NTP efficacy assessment of filamentous fungal spores on seeds, nuts, or peppers. However, from a microbiological standpoint, this methodology is flawed as it relies solely on visual inspection without microscopy.^35^

This research concentrated on assessing the impact of UV-C radiation (254 nm) with varying exposure durations and comparing it with the effect of NTP on four significant foodborne fungal contaminants – *Alternaria alternata*, *Aspergillus niger*, *Fusarium culmorum*, and *Fusarium graminearum*. These fungi are known for their toxin-producing and/or phytopathogenic properties. The influence of these treatments on spore activity under a diverse range of exposure lengths and cell nutritional status was studied.

## Materials and methods

### Microorganisms

All microscopic filamentous fungal strains were obtained from the Collection of Yeasts and Industrial Microorganisms (DBM), University of Chemistry and Technology Prague. Specifically, the following strains were used: *Alternaria alternata* DBM 4004, *Aspergillus niger* DBM 4054, *Fusarium culmorum* DBM 4044, and *Fusarium graminearum* DBM 4344. These fungal strains were inoculated onto potato dextrose agar (PDA, VWR Chemicals, USA). The cultivation was carried out statically at 26 °C for 5 days.^35,36^ The Petri dishes containing the grown cultures were stored at 4 °C for duration not exceeding 1 week.

### Preparation of spore suspension

The fully grown fungal culture containing spores on Petri dish with PDA agar was submerged with a solution of saline (0.9% NaCl (m/v)) to release the spores. Microscopically, the separation of spores only without the presence of vegetative cells was checked. Afterwards, 10 μl of the spore suspension was placed onto a Bürker chamber, and the spore count was determined using light microscopy. A concentration of 10^5^ spores per mL was used for subsequent experiments.^37^ This concentration was achieved by diluting the spore suspension with saline.^35,36^

### UV lamp specifications

A low pressure UV-C lamp (Puritec HNS 30 W G13, Osram, Germany) was used to test the effect of UV radiation (254 nm) on selected fungal strains. The lamp was placed at a distance of 40 cm above the sample, where according to the specification of the UV lamp it had a radiation intensity of 7.75 W m^−2^. UV treatment was carried out with various radiations doses – 60 s (UV dose = 465 J m^−2^), 30 min (UV dose = 13 950 J m^−2^), 60 min (UV dose = 27 900 J m^−2^), 90 min (UV dose = 41 850 J m^−2^).

### NTP generator specifications

The NTP generator was developed within the Department of Physics and Measurements at the University of Chemistry and Technology Prague as described by Khun *et al.*^38^ Six direct current bipolar corona discharges as the sources of NTP generated within a point-to-ring electrode configuration were used. This setup was connected to a high-voltage direct current source. The point electrode was linked to the negative voltage terminal, leading to the initiation of a negative corona discharge at that point. The ring electrode was connected to the positive voltage terminal, resulting in a positive corona discharge on its edge. The NTP discharge generated reactive particles that were subsequently transported from the discharge area, passing through the ring electrode, and were directed onto the microscopic fungal strain located within a well of a microtiter plate. The voltage applied to the discharge electrodes was set at *U* = 6 kV, and each discharge carried a current of *I* = 100 μA corresponding to a bipolar corona discharge. The electric discharge was operated using ambient air at atmospheric pressure as the working gas.

The emission spectrum was dominated by the second positive system of the nitrogen molecule where the vibrational temperature was estimated as *T*_V_ ≈ 3000 K. The first positive system was observed with significantly lower intensity and the emission of both the first negative system of the ionized nitrogen molecule and spectral lines of oxygen atoms were also registered, but its intensity did not exceed a few percent relative to the main emission peak. The emission of hydroxyl radical as well as spectral lines of hydrogen, which are often observed in an air plasma, were not detected due to the relatively low specific power introduced into the discharge plasmas. The detected metastable nitrogen molecules play an important role in plasma kinetics and initiate some plasma-chemical processes outside the discharge region generating ozone, oxygen atoms, and hydroxyl radical that are the species with known high microbicidal effects.

### Monitoring the effect of NTP and UV radiation on microscopic filamentous fungi

**Fig. 1:**
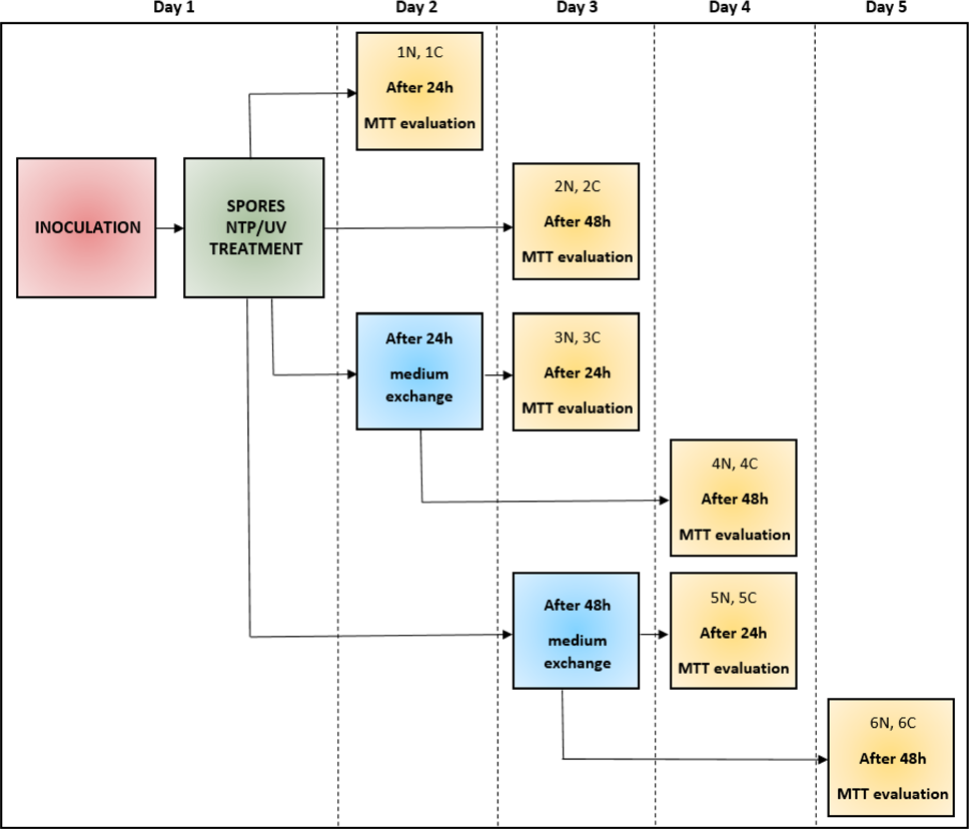
Schematic layout of the experiment with NTP/UV treated spores; green – NTP/UV treatment, blue – PDB medium exchange, yellow – metabolic activity evaluation; N – treated sample, C – control sample.

To study the effect of NTP/UV on spores of filamentous microscopic fungi, sterile, flat-bottomed 24-well polystyrene microtiter plates (TPP, Switzerland) were used. Into each well of the microtiter plate, 1 mL of diluted spore suspension with a concentration of 10^5^ spores per mL was added. Immediately after inoculation of 24-well plates with spore suspension, all plates were exposed to the NTP (90 min) or UV (60 s, 30 min, 60 min or 90 min) treatment. Subsequently, 1 mL of potato dextrose broth (PDB, VWR Chemicals, USA 48 g L^−1^) was added into each well. The plates were cultured statically at 26 °C. The cultivation time depended on the desired length of the experiment. To quantify the resistance of fungal spores to NTP/UV, for each fungal strain 6 different situations were created (each measured in 4 replicates) and the metabolic activity was measured by MTT method. For each measurement, there were always equivalent control wells (non-NTP/UV treated). During the experiment, the original PDB medium was periodically replaced by fresh medium, in order to eliminate the influence of the nutrient limitation on the metabolic activity of cells after spore germination. The entire experiment, during which one strain of filamentous fungi was monitored and evaluated, lasted 5 days.^36^ For clarity, the schematic layout of the experiments is shown in Fig. 1. The absorbance (254 nm) deviation observed in both the pure PDB medium and spore suspension of all four studied filamentous fungi was within a range of up to 5%. Therefore, we neglected UV absorption coefficient in our analysis.^39^

### Metabolic activity measurement

This study concerns exclusively the viability of the spores and the primary metabolism of the fungi considered. The efficiency of NTP/UV on spores of filamentous microscopic fungi was assessed using the MTT (3-[4,5-dimethylthiazol-2-yl]-2,5 diphenyl tetrazolium bromide) assay. MTT is a yellow substance that is reduced by enzymes (mitochondrial dehydrogenases) present in living cells to purple formazan. The intensity of formazan, corresponding to the number of viable cells, can be determined spectrophotometrically. The methodology closely followed the protocol outlined by Kulišová *et al.*^36,37^ After estimated incubation time, the cell suspension was removed from the microtiter plate wells, and the wells were washed twice with phosphate buffer saline (PBS, pH = 7.4). Subsequently, 600 μL of glucose solution (57 g L^−1^ in PBS), 500 μL of MTT solution (Acros Organics, USA, 1 g L^−1^ in PBS), and 150 μL of menadione solution (Merck Life Science, USA, 0.11 g L^−1^ in PBS) were added into each well. The microtiter plate was then incubated on a shaker for 3 hours in the dark (26 °C, 75 rpm). Afterwards, 1 mL of a dissolving solution was added to the wells to dissolve the purple formazan crystals produced as a result of metabolic activity. This dissolving solution consisted of 40% dimethylformamide (v/v) in a 2% acetic acid (v/v) diluted in PBS and 16% sodium dodecyl sulfate (m/v). The plate was again incubated on a shaker for 30 minutes in the dark (26 °C, 150 rpm). Finally, 100 μL from each well was transferred to a 96-well microtiter plate, and the absorbance was measured at 570 nm. A total of 12 replicates (4 parallels in 3 independent repetitions) from each strain were evaluated.

### Preparation of NTP/UV treated spores for SEM

Samples for scanning electron microscopy (SEM) were prepared according to chapter “Monitoring the effect of NTP and UV radiation on microscopic filamentous fungi” and the samples were treated with NTP/UV for 90 minutes. Subsequently, 10 μl of the spore suspension was transferred to an aluminum stub suitable for SEM microscopy. The samples were allowed to dry, and SEM analysis was conducted within 24 hours. Control spore samples, which were not exposed to NTP/UV, were also analyzed by SEM.^36^

### SEM microscopy

In this study, the focused ion beam scanning microscopy (FIB-SEM) instrument (LYRA3, TESCAN GROUP, a.s., Czech Republic) at Research Centre Rez was used. The FIB-SEM system integrates a field electron emission source and a focused ion beam source.

#### SEM imaging of fungal spores

Initially, dehydrated samples of fungal spores underwent a coating process with a 5 nm platinum thin film. The coating was applied at an impact angle of 45° for Pt atoms and under a pressure of 7.10–5 mbar, using a Leica EM ACE600 e-beam coater. The choice of platinum over gold was based on the smaller grain sizes observed in the Pt thin film.

SEM imaging of fungal spores was conducted with an e-beam accelerating voltage of 15 or 20 kV, a current of 465 pA, at working distance of 8–9 mm. Detection of secondary electrons utilized an Everhard–Thornly type detector. Image acquisition parameters included a dwell time of 32 μs per pix and an image resolution of 1024 × 1024 pix, resulting in a frame time of 33.5 s.^35^

#### SEM tomography

The tomographic images were taken on Pt-coated dehydrated spores on Si substrate. Imaging was performed at the accelerating voltage of 15 kV and the current 465 pA at the working distance of 9 mm. The FIB was performed at the accelerating voltage of 30 kV with an emission current of 2 μA and the selected preset for fine milling, polishing. Scanning was performed in automatic mode. A reference cross-shaped mark was milled and used to correct the drift for both FIB and SEM imaging. Reference mark was milled in the vicinity of the object to be imaged. The depth of each slice was set to 3 μm. The scan rate was 32 μs per pix and the image resolution was 1024 × 1024 pix, which gives frame time 33.5 s. FIB slicing and SEM image acquisition were performed through automation.^35^

### Statistical analysis

Statistical analysis was performed in RStudio, where one-way Analysis of Variance (ANOVA) with Tukeyho *post hoc* test and correlation analysis was used for the comparison and evaluation of the metabolic activity measurements. In the case of non-normal distributions, the data were transformed to obtain the normal distributions, using natural logarithm before statistical analysis. Dixon's *Q* test was used for the detection of outliers in data obtained. Four parallel determinations with three independent repetitions were made, ensuring that the deviation between all the measurements was less than 5%.

## Results and discussion

### Effect of UV radiation on fungal spores (expressed by metabolic activity of cells after spore germination)

To establish sterile conditions in food and pharmaceutical industry, UV radiation remains a preferred method in terms of simplicity of execution and safety. Interestingly, despite the challenge of contaminating fungi in the food industry and medicine, there is a lack of publications addressing the effective removal of fungal spores.^6,8^

**Fig. 2:**
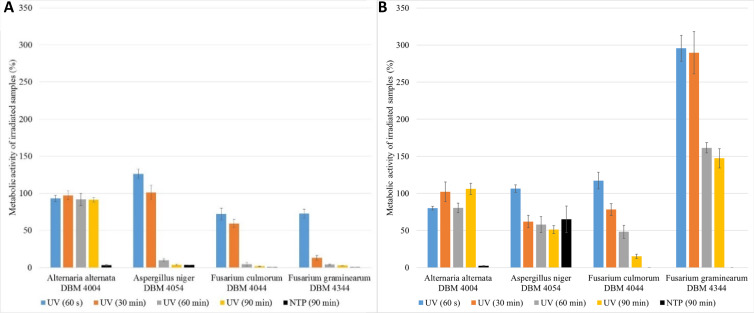
Comparison of the relative metabolic activities of fungi after spores were treated with UV radiation for 60 s (UV dose = 465 J m^−2^), 30 min (UV dose = 13 950 J m^−2^), 60 min (UV dose = 27 900 J m^−2^), 90 min (UV dose = 41 850 J m^−2^) and NTP for 90 min; (A) evaluation after 24 hours after UV/NTP treatment, (B) evaluation after 48 hours after UV/NTP treatment. Metabolic activities were related to situation when spores were not treated with UV/NTP.

None of the articles published so far regarding different light-based approaches effective in the elimination of microorganisms addresses the various adaptation possibilities of treated cells. These publications often overlook the crucial factors of time delay in evaluation of the radiation's effects and subsequent culture conditions.^24,40^ In this work a comprehensive approach was used to evaluate the impact of UV treatment durations (60 s (UV dose = 465 J m^−2^), 30 min (UV dose = 13 950 J m^−2^), 60 min (UV dose = 27 900 J m^−2^), 90 min (UV dose = 41 850 J m^−2^)) on metabolic activities of the cells 24 and 48 hours after treatment. Given the substantial number of experiments conducted (6 for each filamentous fungus and one radiation time, in total 96 experiments, for clarity see Fig. 1), we present a selected subset of results. Fig. 2 shows the relative metabolic activities of fungi after different doses of UV radiation on fungal spores, both 24 and 48 hours after treatment, where the metabolic activities were related to fungi without UV/NTP treatment. In none of the studied situations did UV radiation have a 100% effect against irradiated fungal spores. Even after a 90 minutes exposure to UV radiation, there was no complete inhibition of spore germination. The efficacy of fungal spore destruction depends on the duration of UV exposure, the total dose, the environmental conditions (water activity, nutrient availability), and the specific microorganism type.^6^ For several decades, 254 nm UV light has been employed as a disinfectant tool against fungal spores, leveraging DNA disruption.^41^ Studies have documented the successful use of UV-C radiation (100–280 nm) in decontaminating bacterial vegetative spores of *Alicyclobacillus acidoterrestris*.^42^ Nakpan *et al.* reported that bacterial spores exhibit an order of magnitude greater sensitivity to UV radiation compared to *Aspergillus fumigatus* spores.^20^

As depicted in Fig. 2, *A. alternata* spores exhibited the least susceptibility to UV radiation. Even after 90 min irradiation, these spores germinated, and metabolic activity of cells after spore germination after 24 hours after treatment remained equivalent to the control samples across all radiation doses (in range 91.3–97.3% compared to control samples). Wang *et al.* also demonstrated the increased resistance of *A. alternata* to UV radiation.^43^ On the other hand, UV radiation demonstrated its highest efficacy on *Fusarium* sp. spores, where higher doses of radiation lead to a metabolic activity reduction exceeding 95% compared to the control. Fig. 2B illustrates the irradiated spores and measured metabolic activity of cells after spore germination of measured 48 hours after the treatment. In the case of *F. graminearum*, a significant increase in metabolic activity was observed after irradiation, surpassing the control at all radiation doses. Other studies also report increased resistance of *F. graminearum* to UV radiation,^44–46^ even compared to *F. culmorum*.^47^ For *A. niger* and *F. culmorum* exposed to 90 min UV treatment, the relative metabolic activities increased to 50% and 15%, respectively (Fig. 2B). In Fig. 2, the columns depicting relative metabolic activities after 90 minutes of NTP exposure reveals nearly a 100% impact in all monitored scenarios, with the exception of the microorganism *A. niger* when measured after 48 hours.

The results clearly indicate that the effective UV radiation dose depends not only on the type of filamentous fungi but also on the time between UV exposure and the evaluation of metabolic activity. For bacteria such as *Escherichia coli*, *Salmonella typhimurium*, and *Listeria monocytogenes*, a reported effective UV-C radiation dose is 400 J m^−2^,^48^ which is lower than the minimum dose tested in this study, namely 60 s, (465 J m^−2^). By comparing the efficacy of UV radiation on bacteria and filamentous fungi it was found that the effective dose for medical bacteria (*Streptococcus* and *Staphylococcus*) was 60 J m^−2^, while for microorganisms causing food spoilage (bacteria, yeasts, sporulating filamentous fungi), the effective dose (D^90^) was 387 J m^−2^ for bacteria (3 minutes treatment) and 4128 J m^−2^ for filamentous fungi (32 minutes treatment).^27^ The maximal radiation dose employed in this work was 41 850 J m^−2^ and even this dose proved insufficient to completely inactivate fungal spores, when measured after 24 and 48 h of germination.

Challenges associated with the use of a fixed UV source include the adaptive mechanisms of fungi at both molecular and cellular levels, as well as spore pigmentation that absorbs UV light at similar wavelengths, providing protection against its effects.^49^ Despite the not very satisfactory results of UV treatment on spores of filamentous fungi, it can be stated that employing UV radiation, as one of the dry sanitation methods, has the potential to support the efficacy of conventional approaches while maintaining environmental friendliness by generating no waste.^10,11^ However, the use of UV radiation as the sole sanitizing method would be ineffective in maintaining a sufficient sanitizing process due to the high rate of active spores capable of growth after UV treatment.

**Fig. 3:**
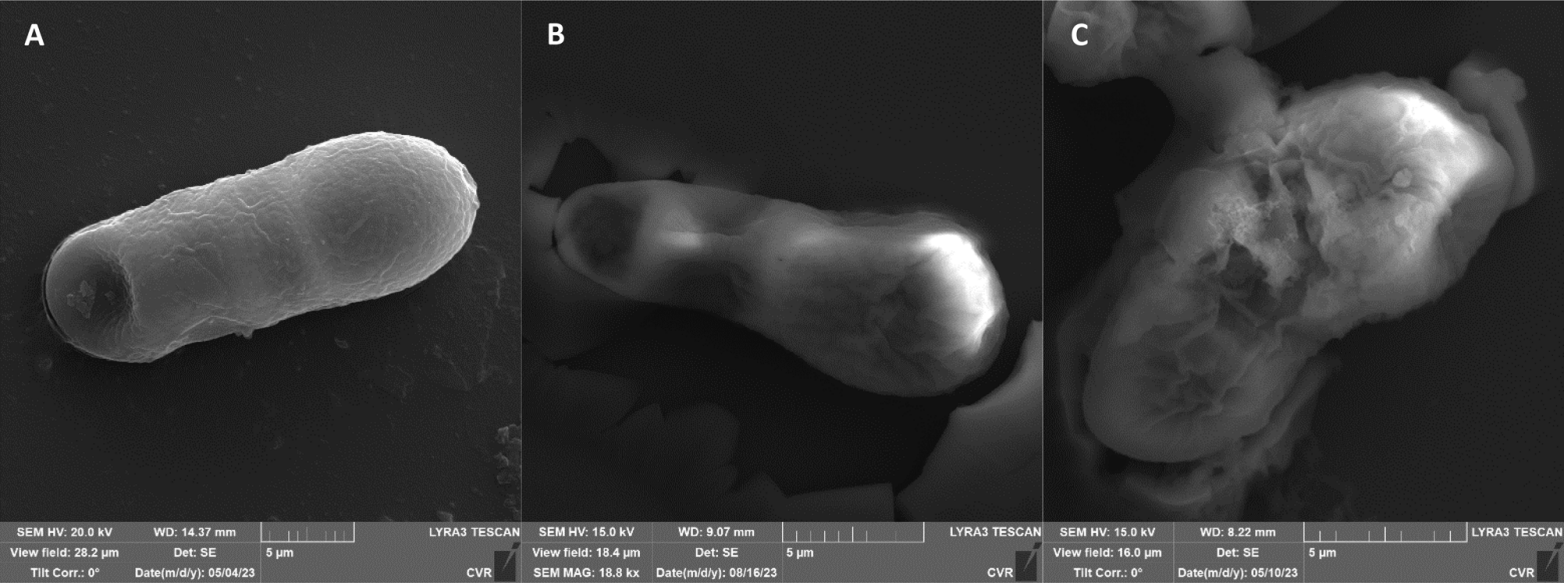
*A. alternata* DBM 4004 spores ((A) control, (B) after UV radiation, (C) after NTP).

**Fig. 4:**
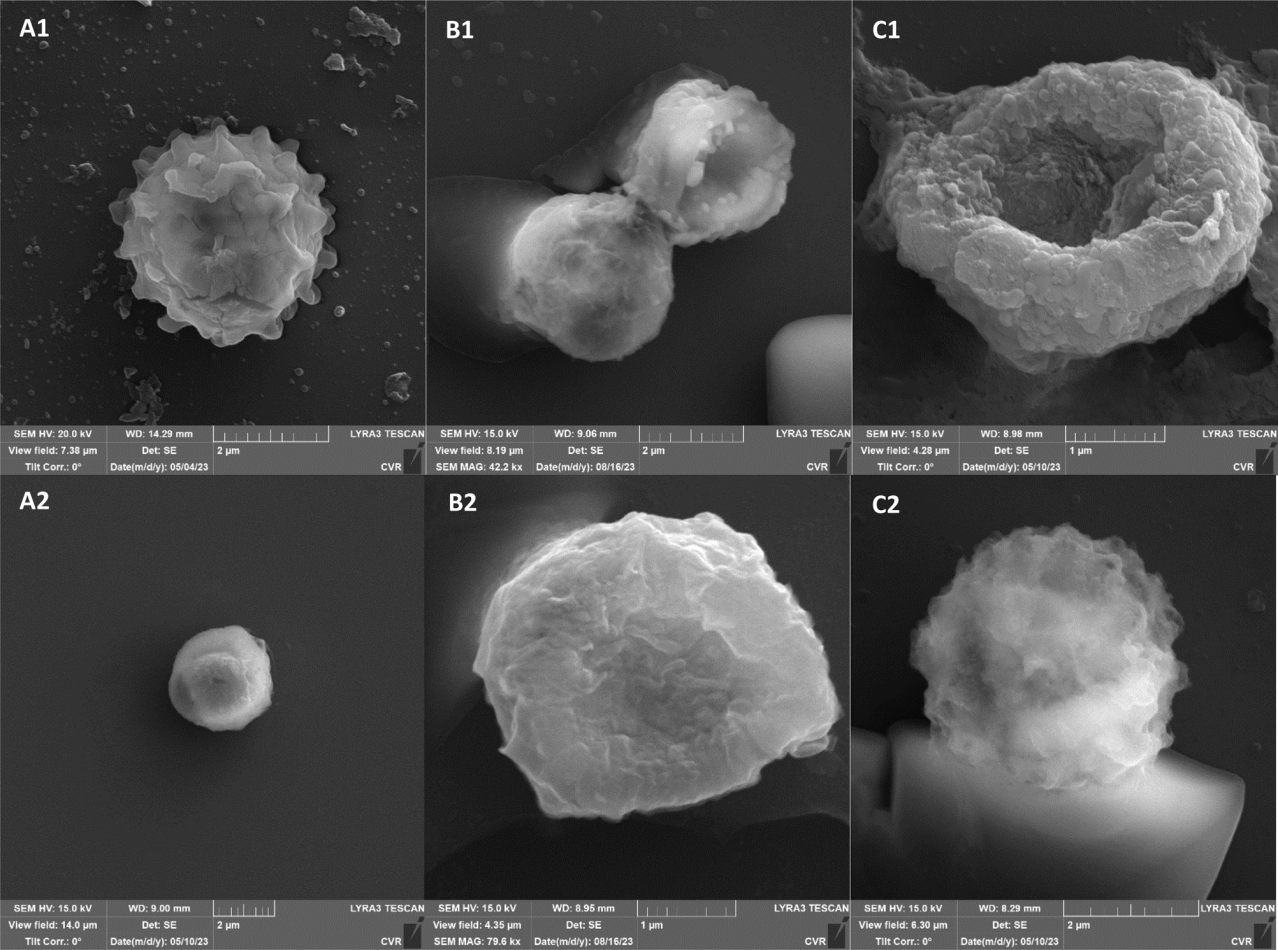
*A. niger* DBM 4054 spores ((A) control, (B) after UV radiation, (C) after NTP).

**Fig. 5:**
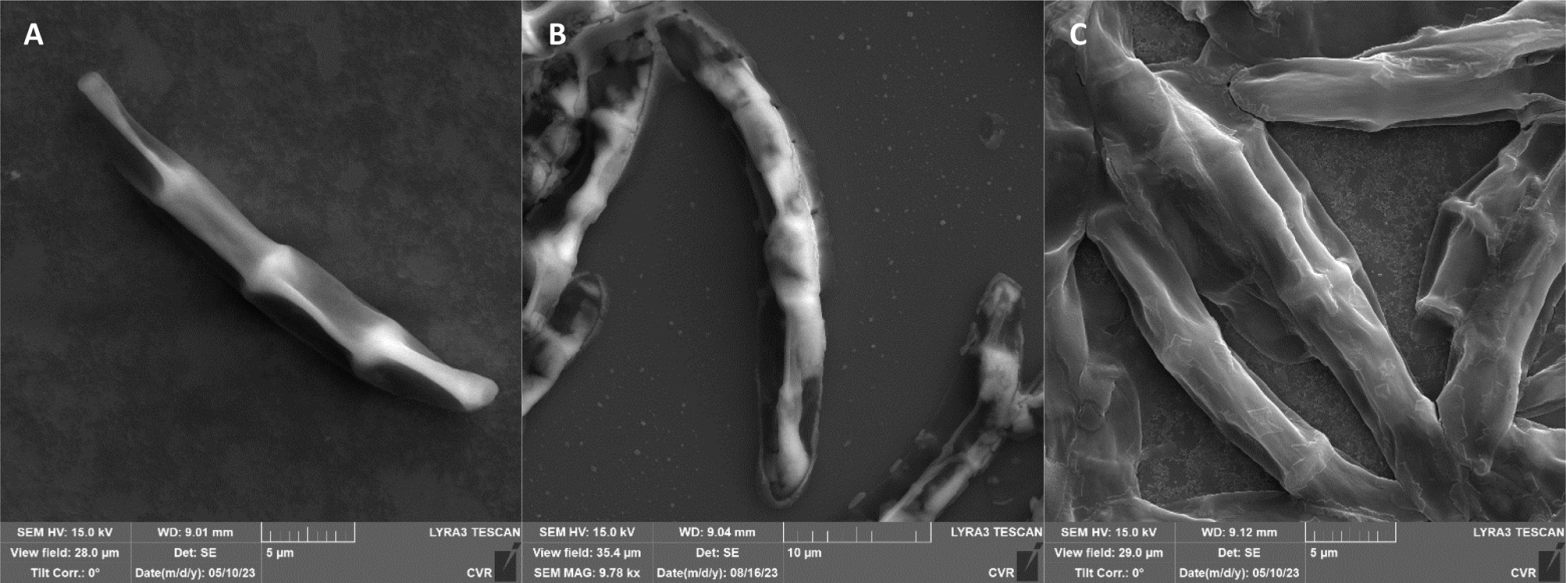
*F. culmorum* DBM 4044 spores ((A) control, (B) after UV radiation, (C) after NTP).

**Fig. 6:**
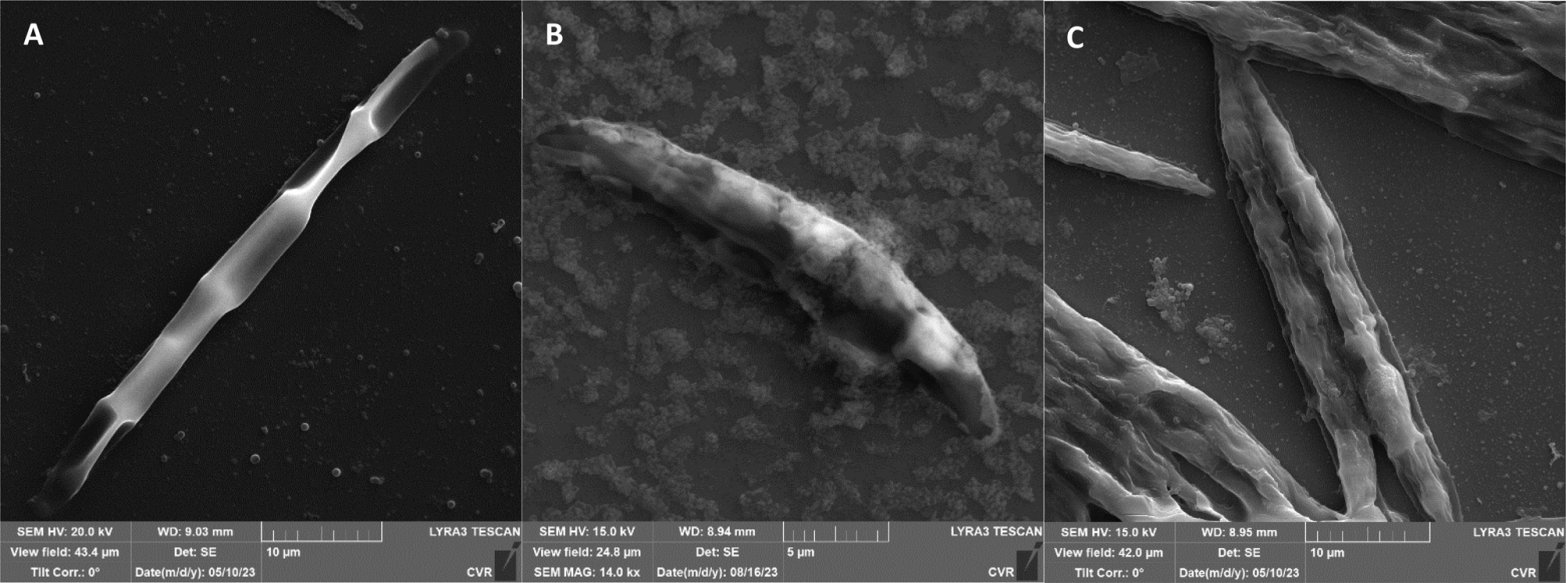
*F. graminearum* DBM 4344 spores ((A) control, (B) after UV radiation, (C) after NTP).

The impact of UV radiation on filamentous microscopic fungi was also visually documented through SEM images. The spore size was reduced in case of spores of *A. alternata* and *F. graminearum* after UV treatment (Fig. 3 and 6), whereas the spores of *A. niger* and *F. culmorum* retained their original size (Fig. 4 and 5). Some studies indicate the potential emergence of microorganism resistance to UV radiation. Davies *et al.* and Goldman *et al.* reported the development of increased tolerance in *E. coli* cells following repeated UV radiation doses, likely attributed to mutations in genes responsible for DNA repair and replication. Cells exhibiting increased resistance to UV radiation were noted to have enlarged cell size, considered a possible mechanism for reducing the lethal effects of UV radiation on cells.^50–52^ Additionally, after UV exposure there is a noticeable thinning of cell walls, particularly in *Fusarium* strains (Fig. 5B and 6B), accompanied by the condensation of cell contents.

### Comparison of the effect of UV radiation and NTP on microscopic filamentous fungi

Since UV radiation was not completely effective on the selected strains of filamentous fungi even after a 90 minutes irradiation, an experiment investigating the impact of NTP on fungal spores was conducted. Although the exact mechanism of NTP action on microorganisms is not fully understood, it is recognized that the excitation of a plasma discharge produces reactive oxygen species (ROS) and reactive nitrogen species (RNS) through collisions and energy exchange between electrons, atoms, and neutral gas molecules.^11,53^ Preliminary studies suggest that the microbicidal effect of NTP results from oxidative stress on cells induced by reactive species, leading to membrane lysis, DNA damage, and dehydration of lipids, proteins, and cells.^53–55^ There is also evidence of a synergistic effect between oxygen atoms and UV photons generated by the gaseous plasma.^56^ Plasma likely inactivates spores through corrosion caused by oxygen atoms, radicals, and its own photodesorption, disrupting the protective layers of spores.^57,58^

A comprehensive examination of the impact of NTP on filamentous fungi spores can be found in the article by Hoppanová *et al.*^34^ Despite numerous studies reporting a notable decrease in viable spore numbers following NTP application for durations ranging from 10 to 45 minutes, achieving complete spore inactivation (100%) has not been observed.^59–63^ These referenced publications primarily focus on assessing NTP efficacy through the enumeration of colony-forming units (CFU) and determination of *D*-values. It is noteworthy, however, that such expressions are tailored for unicellular organisms like bacteria and yeast.

The *D*-value is commonly employed for assessing single-celled organisms, including the characterization of bacterial endospores. These endospores, upon transitioning into the metabolically active phase, remain as single-celled entities. However, filamentous fungi's asexual spores undergo a transformation from dormancy to hyphal formation, rendering them unsuitable for evaluation in terms of CFU.

All experimental variants were exposed to UV or NTP discharge for 90 minutes. As indicated in the subsequent graphs (Fig. 8), NTP demonstrated high effectiveness, nearly 100%, against spores of *A. alternata*, *F. culmorum*, and *F. graminearum*. The relative metabolic activities after exposure to NTP discharge were in the order of percent units, while after exposure to UV radiation, the values were significantly higher. Only *A. niger* spores exhibited greater resistance compared to the other strains, with no significant difference in efficacy between UV and NTP (Fig. 8). The smaller impact of both types of radiation on *A. niger* spores can be attributed to pigmentation of spores of this genus, as it is known that radiation is partially absorbed, thereby reducing its effect.^49,64^ Molina-Hernandez *et al.* also note the high resistance of *A. chevalieri* spores to NTP radiation, with only a 32% inhibition of germination after 30 minutes.^65^

SEM images supporting the metabolic activity results depict a comparison between control spores and those treated with UV and NTP (Fig. 3–6).

Additional set of photos of *A. niger* spores were included due to increased variability in the visual characteristics of both control and treated spores. In the case of the other strains, SEM analysis revealed consistent appearances for control spores, and the alterations in morphology after treatment were remarkably similar across these strains as well. In Fig. 4, it is possible to observe changes in the surface of spore of *A. niger* after the action of NTP, when the surface and structures of the spore were significantly disturbed, up to its “falling off” (Fig. 4C(1)). In the second variation of morphological changes following NTP treatment, no significant alteration in the spore shape was evident; the spore retained its globular form with notable protrusions (Fig. 4C(1)) akin to a first type of control spores (Fig. 4A(1)). Smooth-structured spores were also observed among control specimens (Fig. 4A(2)). After exposure to UV, cell wall collapse was noticeable, although it was not as distinct as the disruption observed after exposure to NTP (Fig. 4B(2)). Ki *et al.* made similar images and observations with spores of *Aspergillus brasiliensis*. They treated spores with NTP for 3 min and it caused the disruption of the cell wall structure, which is an essential factor in maintaining the shape and integrity of the fungal cell. NTP was found to induce the loss of cell wall integrity and its protective role in spores. As cell membranes control traffic into and out of cellular components, if these membranes are damaged, they lose their regulatory function, potentially leading to the leakage of intracellular components like nucleic acids, proteins, and other metabolites.^66^ Distinct wrinkling and thinning of the cell walls are evident in the spores of both *Fusarium* strains following NTP treatment, whereas control spores exhibit a smooth surface without significant irregularities.

**Fig. 7:**
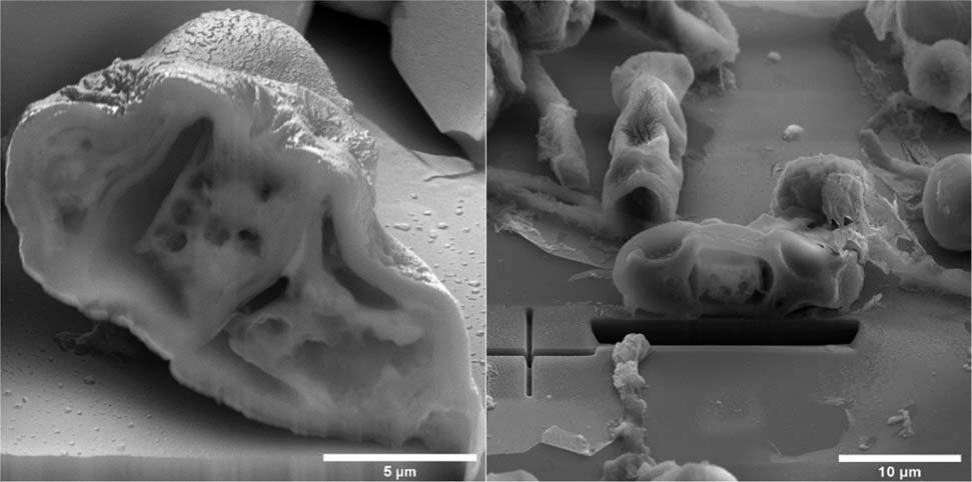
*A. alternata* DBM 4004 spore sections after NTP treatment.

On spore sections of *A. alternata* is evident that the spores have distinct chromatin condensation, massive disruption of the cytoplasmic matrix and cytoplasmic vacuolization after NTP treatment (Fig. 7). Previous works describe a similar impact of NTP treatment on *Aspergillus flavus* spores, highlighting intracellular destruction, the induction of intracellular ROS and NO accumulation, disruption of the antioxidant and energy metabolic systems leading to apoptosis due to mitochondrial dysfunction, and a decrease in reduced forms of glutathione along with lipid peroxidation.^34,65,67,68^ Simultaneously, some studies revealed that NTP either reduced the production of aflatoxin B1 (ref. 67 and 69) or reported the high efficacy of NTP in removing fungal mycotoxins.^70^ The varying effectiveness of NTP on different fungal representatives may be linked to their robust polysaccharide cell walls, providing protection against environmental stress.^71,72^ Additionally, the erosion of the cell wall due to the action of charged particles can disrupt its integrity and thickness.^73^

**Fig. 8:**
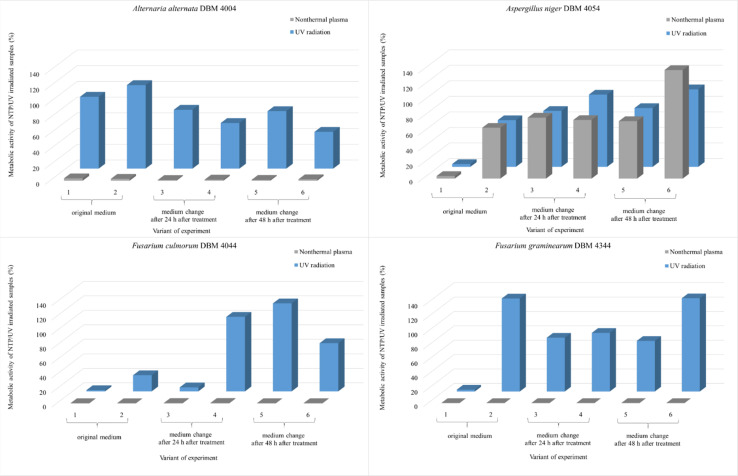
Comparison of the effect of UV radiation and NTP (90 min treatment) on the spores of the filamentous fungi *A. alternata* DBM 4004, *A. niger* DBM 4054, *F. culmorum* DBM 4044, *F. graminearum* DBM 4344. Within each of the six variants, the evaluation took place after 24 and 48 hours after the treatment of the sample with the original PDB medium (1st and 2nd variant), for the 3rd and 4th variant, the PDB medium was exchanged after 24 hours and the evaluation took place after 24 and 48 hours after the exchange, for the 5th and 6th variants, the PDB medium was exchanged after 48 hours and the evaluation took place after 24 and 48 hours after the exchange (see Fig. 1).

There is a scarcity of studies investigating the impact of NTP on metabolic activity of filamentous fungi. In one of them, Sakudo *et al.* conducted experiments where *Aspergillus brasiliensis* cells were exposed to NTP for 5 minutes. After this time period, cell viability remained unaffected, but after 15 minutes, a notable impact was observed.^74^ Among the four filamentous fungi examined in our study, *A. niger* demonstrated the highest resistance to the effects of both UV and NTP radiation when evaluating metabolic activity after treatment (Fig. 8). Soušková *et al.* also noted in their research that among the fungi they investigated (including *Aspergillus oryzae*, *Cladosporium sphaerospermum*, and *Penicillium crustosum*), *A. oryzae* exhibited the greatest resistance to the effects of NTP.^21^

When studying the effect of potential disinfectants, it is also necessary to take into account the time after which the metabolic activity is evaluated, whether nutrients from the medium could have been depleted. The only study addressing the impact of UV radiation on cells and subsequent new supply of nutrients was conducted by MeiTing *et al.* on bacteria. According to their findings, the effect of UV radiation on *E. coli* in wastewater decreased after the addition of a new batch of wastewater, the number of *E. coli* increased from the initial inoculation of 4 × 10^3^ to 9 × 10^3^ CFU mL^−1^ after a 72 hours incubation period. In the case of *Bacillus subtilis*, which forms resistant endospores, its growth was unaffected by the addition of new nutrients.^75^ These findings show not only the higher resistance of spore producing bacteria to radiation but also highlights the importance of monitoring the potential influence of nutrient sources. If we follow our results, we do not observe a clear trend after the addition of a new medium for any of the monitored microorganisms. The supply of new nutrients therefore had no significant effect on the change in metabolic activity.

It is interesting that none of the articles focused on the comparison of the effects of UV radiation and NTP on microorganisms compares these two types of radiation from a physical perspective.^7,18,24,32^ Since the geometrical arrangement of the experiment using a UV-C low pressure lamp and NTP (transient spark) is significantly different, comparing the efficiency of the two devices in terms of performance is problematic. While the active area of the UV lamp could potentially be larger, the area of action of the NTP cannot be extended much further. The comparison of efficiency must therefore always be related to the specific situation.

The potent microbicidal efficacy of NTP has been studied for two decades but is still awaiting widespread commercial application.^76^ Researchers are optimistic about the potential successful integration of NTP into food processes, if the knowledge obtained in the literature so far is accurately interpreted. It is crucial to consider not only the inactivation of bacteria or yeast but also more resistant forms of microorganisms, including bacterial endospores and filamentous fungi and their spores. This study stands out as the first of its kind, providing a detailed comparison of the efficacy of UV radiation and NTP on spores and their development in time of four major filamentous fungal food contaminants. It assesses the evident effectiveness of NTP radiation, regardless of additional nutrient supply to fungal cells.

## Conclusions

In conclusion, our comprehensive investigation into the efficacy of UV radiation and NTP on filamentous fungi spores, specifically *Alternaria alternata*, *Aspergillus niger*, *Fusarium culmorum*, and *Fusarium graminearum*, has yielded significant insights. Fungal contamination poses persistent challenges in various industries, prompting the exploration of innovative antimicrobial strategies. While UV radiation, a conventional method, showcased variable efficacy, NTP emerged as a highly promising alternative, achieving nearly 100% effectiveness against fungal spores. UV radiation, despite being a common sterilization method, did not achieve complete inhibition of microbial growth. SEM analysis showed morphological changes in structure after both treatments. NTP exposure resulted in significant damage to the cell wall and in multiple cases to complete collapse of the cell structure.

Importantly, the study contributes to the literature by highlighting the limitations of UV radiation and the potential of NTP for eliminating filamentous fungi. The complex connections of factors such as radiation dose, fungal species, and exposure duration highlight the need for comprehensive approaches in fungal decontamination. Our findings support the view that NTP exhibits higher inhibition efficiency, making it a promising candidate for enhanced sanitation processes in diverse industries. In the evolving landscape of antimicrobial strategies, further research in NTP area is needed, particularly in addressing challenges posed by filamentous fungi, ultimately paving the way for more effective and environmentally friendly sanitation processes across industries.

## Author contributions

Markéta Kulišová: conceptualization, methodology, investigation, writing – original draft, writing – review & editing; Michaela Rabochová: methodology, investigation, formal analysis; Jan Lorinčík: data curation, methodology; Olga Maťátková: writing – review & editing; Tomáš Brányik: methodology, formal analysis, resources, supervision, funding acquisition; Jan Hrudka: methodology, investigation; Vladimír Scholtz: data curation, validation; Irena Jarošová Kolouchová: conceptualization, resources, data curation, supervision, funding acquisition.

## Conflicts of interest

There are no conflicts to declare.

## Supplementary Material
